# Machine learning-based predictive model for high- grade cytokine release syndrome in chimeric antigen receptor T-cell therapy

**DOI:** 10.3389/fimmu.2025.1692892

**Published:** 2025-11-20

**Authors:** Xiaofeng Yu, Qingqing Wang, Tangnuran Halimulati, Jiling Lv, Kai Zhou, Guilai Chen, Li Yin, Yulin Liu, Jingwang Bi, Zhuo Xiang, Qiang Wang

**Affiliations:** 1 Clinical Medicine Research Center, Shandong Second Provincial General Hospital, Jinan, China; 2 Department of Respiratory Medicine, Shandong Second Provincial General Hospital, Jinan, China; 3 Department of General Surgery, The First Affiliated Hospital, Army Medical University, Chongqing, China

**Keywords:** CAR-T therapy, cytokine release syndrome, COVID-19, machine learning technique, XGBoost model

## Abstract

**Introduction:**

The development of robust predictive models for high-grade cytokine release syndrome (CRS) in CAR-T recipients remains limited by sparse clinical trial data.

**Methods:**

We analyzed of 496 COVID-19 patients revealed that CRS plays a pivotal role in disease progression and serves as a valuable data source for understanding CRS progression. Building on this insight, we evaluated and compared the predictive performance of three machine learning models, with the ultimate goal of developing a predictive model for high-grade CRS in patients receiving CAR-T therapy.

**Results:**

Among evaluated algorithms (XGBoost, Random Forest, Logistic Regression), XGBoost demonstrated superior performance in high-grade CRS prediction. Feature importance analysis identified SpO2, D-dimer, diastolic blood pressure, and INR as key predictors, enabling development of a validated riskassessment algorithm. In an independent CAR-T cohort (n=45), the algorithm achieved impressive predictive performance for high-grade CRS prediction.

**Discussion:**

Using machine learning, we identified key clinical biomarkers strongly associated with high-grade CRS. This tool efficiently predicts progression to high-grade CRS post-onset and shows significant potential for clinical deployment in CAR-T therapy.

## 1 Introduction

Chimeric antigen receptor T-cell (CAR-T) therapy has made significant advances in the treatment of malignant tumors. However, the risk of severe adverse events, particularly cytokine release syndrome (CRS), can lead to life-threatening complications (1). High-grade CRS severely limits the safety and efficacy of CAR-T therapy, making early detection and intervention crucial. Previous studies have suggested that factors such as CAR-T cell expansion dynamics, tumor burden, and baseline lymphocyte counts can predict the severity of CRS, underscoring the need for effective monitoring systems (2). However, the complexity of existing monitoring systems has restricted their clinical application. Therefore, it is imperative to develop an early predictive system for high-grade CRS using clinical observational data from CAR-T treatments. However, due to the scarcity of large-scale clinical trials in CAR-T treatment, an early predictive model based on such data has not yet been realized.

High-grade CRS is not only a complication frequently encountered in the context of tumor immunotherapies, but also commonly observed in patients with COVID-19. Despite differences in the pathogenesis, CRS patients present with similar symptoms and signs, CAR-T therapy related CRS primarily arises from the anti-tumor immune response, whereas in COVID-19, CRS is precipitated by viral infection and the resulting immune dysregulation (3). However, immune activation and inflammatory responses play a central role in the immune response to both diseases. The occurrence of severe CRS in COVID-19 patients is often accompanied by a cascade of serious complications and increased mortality (3, 4). Notably, the exacerbation in the late stages of COVID-19 has been found to correlate with specific biomarkers of CRS, including interleukin-1β (IL-1β) and tumor necrosis factor-α (TNF-α) (5). These clinical observations in COVID-19 patients provide a valuable dataset for the understanding for high-grade CRS.

Machine learning models have achieved significant breakthroughs in medical research and clinical practice, particularly in predicting disease progression. By leveraging large datasets and advanced algorithms, machine learning models can identify patterns that offer more precise and personalized predictive analyses, thereby optimizing clinical decision-making and patient management. For instance, support vector machines and boosted decision trees have notably enhanced the accuracy of predicting disease progression and prognosis in conditions such as cancer and diabetic nephropathy, through the analysis of complex and heterogeneous data (6, 7). This study aims to apply machine learning models to explore a predictive model for high-grade CRS in tumor patients undergoing CAR-T therapy.

## 2 Materials and methods

### 2.1 Data collection and clinical assessment

A retrospective observational study of COVID-19 was conducted in two cohorts: Wuhan Taikang Hospital (Wuhan, China) between December 2019 and April 2020, and Shandong Second Provincial General Hospital (Jinan, China) between December 2022 and February 2023. All enrolled patients developed cytokine release syndrome (CRS) and had complete clinical data, including demographics, presenting symptoms at admission, disease course, and laboratory values obtained during hospitalization. Baseline information and laboratory results were collected from the electronic medical record systems of both hospitals.

The collected variables included age, gender, and medical history (cardiovascular disease, diabetes mellitus, chronic lung disease, chronic kidney disease, chronic liver disease, cerebrovascular disease, and malignant tumor); respiratory rate (RR), heart rate (HtR), oxygen saturation (SpO_2_), systolic and diastolic pressure, oxygen inhalation mode, coagulation parameters (prothrombin time [PT], international normalized ratio [INR], thrombin time [TT], activated partial thromboplastin time [APTT], fibrinogen [FIB], fibrinogen degradation products [FDP], and D-dimer), hospital stay, ICU admission, and clinical outcomes (improved or aggravated). Coagulation and other monitoring parameters were collected at least twice: initially at the onset of CRS and subsequently during the convalescent phase or upon progression to the highest severity grade. CRS diagnosis and grading were performed according to CTCAE 5.0 (**Supplementary Table S1**). Patients were stratified into two groups: Low-grade CRS (Grades 1–2) and High-grade CRS (Grades 3–5).

In the COVID-19 cohorts, participants were included if they met the following criteria: ① aged ≥18 years and treated according to the national COVID-19 management guidelines; ② tested positive for SARS-CoV by PCR or serology, with radiologic evidence of pneumonia on chest CT; ③ patients were diagnosed with CRS by at least two clinicians. ④ patients suspected of COVID-19 or pregnant women were excluded.

In the CAR-T therapy cohort, 45 consecutive patients were enrolled at Shandong Second Provincial General Hospital between August 2021 and September 2024. Baseline characteristics and CRS clinical trajectories were recorded during the observation period following CAR-T infusion (Days 0–14). Monitoring data were collected at multiple time points (Days 4, 7, and 10), including four clinical parameters: SpO_2_, D-dimer, diastolic pressure, and INR.

We excluded cases with incomplete monitoring data and collected clinical data from a total of 25 CRS patients, including biomarker levels at CRS onset and sequential timepoints (days 4, 7, and 10). A validation cohort consisting of 36 complete records (6 high-grade CRS and 30 low-grade CRS) was used as the test set for evaluating the predictive model of high-grade CRS risk. Each record was annotated with CRS grade contemporaneous at sampling timepoints. The cohort data were processed through the predictive model for computational validation and results were compared with clinical outcomes.

The inclusion criteria of CAR-T therapy cohort were as follows: ① patients with advanced, metastatic, or relapsed malignancies confirmed by immunohistochemistry (IHC) to express the target antigen; ② those who had failed at least second-line standard therapy or lacked effective treatment options; ③ patients with at least one measurable lesion; ④ Eastern Cooperative Oncology Group (ECOG) score of 0–2. Exclusion criteria included patients with central nervous system metastases, uncontrolled systemic infections, or significant dysfunction of vital organs.

This study was approved by the Ethics Committee of Shandong Second Provincial General Hospital (Q-2023037, LS-2021-008-001, LS-2021-009-001, and LS-2022-004-001). Detailed inclusion and exclusion criteria for both studies are provided in the Supplemental Eligibility Criteria.

### 2.2 Machine learning model development and validation

We developed and validated a machine learning model to predict high-grade CRS using clinical observational data from 496 COVID-19 patients diagnosed with CRS. The overall analytical workflow included data preprocessing, imputation of missing values, model construction, hyperparameter optimization, and performance validation. Missing data were imputed using the non-parametric missForest algorithm (v1.4) (8), with iterative imputations repeated until the prediction error stabilized, ensuring reliable and accurate estimation.

After imputation, the complete dataset was randomly divided into an 80% training set and a 20% testing set, with subgroup stratification based on median values and clinically relevant criteria. Univariate Cox regression analyses were conducted to identify prognostic variables associated with disease aggravation, and hazard ratios (HRs) with 95% confidence intervals (CIs) were calculated to quantify their impact. Subsequently, three machine learning algorithms—Lasso regression, Random Forest, and Extreme Gradient Boosting (XGBoost)—were applied to construct predictive models. Hyperparameters were optimized using GridSearchCV (9, 10) with cross-validation to ensure optimal performance. Among these, XGBoost demonstrated the best predictive capacity for high-grade CRS. To enhance interpretability, SHapley Additive exPlanations (SHAP) (11) were used to quantify the contribution of each feature to model predictions, while Receiver Operating Characteristic (ROC) curve analysis was employed to assess model discrimination and determine optimal cutoffs via the Youden Index. The final model achieved high accuracy, sensitivity, and area under the curve (AUC) values, and its predictive robustness was further validated using clinical data from 45 cancer patients receiving CAR-T therapy.

#### 2.2.1 Dataset construction using missForest for imputation of missing data

Missing data were imputed using the missForest package (version 1.4) (8). This non-parametric algorithm applies random forest regression trees for continuous variables and classification trees for categorical variables. Missing values were iteratively predicted, with each variable imputed using all other available variables as predictors. The iterative process continued until the imputation error stabilized or reached the preset maximum number of iterations, evaluated using normalized root mean squared error (NRMSE) for continuous variables and proportion of falsely classified entries (PFC) for categorical variables. To balance accuracy and computational efficiency, each forest contained approximately 100 trees, and the maximum number of iterations was limited to 10, ensuring stable and reliable estimates. After data imputation, the complete dataset was randomly divided into a training set (80%) and a testing set (20%), with variables further stratified according to median values and clinically relevant classification criteria to enable subgroup analyses while maintaining statistical robustness and clinical interpretability.

#### 2.2.2 Forest plot analysis

To comprehensively explore subgroup characteristics and identify predictive factors associated with disease aggravation or other clinical outcomes, univariate Cox regression analyses were performed within each predefined subgroup (12, 13). The pooled dataset was stratified by median values or established clinical criteria, ensuring adequate representation across subgroups and maintaining both statistical robustness and clinical interpretability.

Univariate Cox regression was conducted using the R survival package (v3.5-5). Hazard ratios (HRs) were calculated as the exponential of the regression coefficient, and 95% confidence intervals (CIs) were derived using the Wald test. Statistical significance was determined at a two-sided P < 0.05.

The baseline variables included in subgroup analyses were age, gender, tumor stage, comorbidities (e.g., cardiovascular disease, diabetes mellitus, chronic lung disease, chronic kidney disease), and selected laboratory markers. Variables with missing data were imputed using the missForest algorithm described previously. Detailed subgroup stratification criteria and the complete list of baseline variables are provided in **Supplementary Table S2**.

Results were visualized using forest plots generated with the forestplot (v3.1.1) and ggplot2 (v3.4.2) packages, where each horizontal bar represents the HR and its corresponding 95% CI. These visualizations provided an intuitive representation of effect sizes across different subgroups, facilitating the identification of high-risk factors within clinically meaningful patient strata.

#### 2.2.3 Construction and evaluation of machine learning models

Three machine learning algorithms were employed to construct predictive models: Lasso regression, Random Forest, and Extreme Gradient Boosting (XGBoost). For each model, hyperparameter optimization was conducted using GridSearchCV with cross-validation to ensure fair comparison under optimal configurations.

Lasso regression (13, 14):

Lasso regression, incorporating L1 regularization, was used to reduce model complexity and handle high-dimensional features. Hyperparameter tuning was performed for the regularization coefficient (α, ranging from 0.001 to 1). The optimal α was selected via 10-fold cross-validation, minimizing prediction error while avoiding over-penalization.

Random Forest (13, 15):

Random Forest, based on an ensemble of decision trees, improved robustness and reduced overfitting. Key hyperparameters tuned included:

- Number of trees (n_estimators): 100–500
- Maximum tree depth (max_depth): 3–10
- Minimum samples per split (min_samples_split): 2–10

Maximum features per split (max_features): √p or $log_{2}(p)$ The optimal model was obtained with approximately 300 trees and a maximum depth of 6, achieving a balance between variance and bias.

XGBoost (13, 16):

XGBoost, a gradient boosting algorithm, iteratively minimized prediction errors and effectively captured nonlinear relationships. The final optimized hyperparameters were as follows:

- learning_rate = 0.05
- n_estimators = 100
- max_depth = 4
- min_child_weight = 3
- subsample = 0.8
- colsample_bytree = 0.8
- gamma = 0.1
- lambda = 1.5
- alpha = 0.1

This configuration maximized predictive performance while controlling model complexity.

#### 2.2.4 SHAP analysis and ROC curve evaluation

To improve the interpretability of the XGBoost model, SHapley Additive Explanations (SHAP) were applied to quantify and visualize the contribution of individual features to model predictions. SHAP values were computed using the Python SHAP package (v0.41.0). Each feature’s contribution to the prediction was quantified as follows (13, 17):

- Positive SHAP values indicate that a feature increases the probability of high-grade CRS.
- Negative SHAP values indicate that a feature decreases the probability of high-grade CRS.
- Visualization methods included:
- SHAP summary plots, displaying overall feature importance across all samples.
- SHAP dependence plots, illustrating the marginal effect of individual features.
- Feature importance bar plots, ranking predictors according to their mean absolute SHAP values.

This approach allowed identification of the most influential clinical factors driving high-grade CRS risk and enhanced the model’s mechanistic interpretability.

Additionally, Receiver Operating Characteristic (ROC) curves were generated using the scikit-learn library (v1.2.2) to evaluate classification performance across thresholds (13). For each threshold, the True Positive Rate (TPR) and False Positive Rate (FPR) were calculated as:


TPR=TP/(TP+FN)

FPR=FP/(FP+TN)The Area Under the Curve (AUC) served as the primary performance metric, representing global model discrimination ability. Bootstrapping (n = 1000 resamples) was conducted to estimate 95% confidence intervals of AUC, and the optimal classification threshold was determined by maximizing the Youden index (TPR – FPR).

Together, SHAP and ROC analyses provided both mechanistic interpretability and robust performance validation, ensuring that the XGBoost model achieved both predictive accuracy and clinical transparency.

#### 2.2.6 Determination of optimal cutoff and Youden Index calculation

To determine the optimal classification threshold for the model, Receiver Operating Characteristic (ROC) curve analysis was performed on the training dataset (13). For each potential cutoff value, sensitivity and specificity were calculated, and the Youden Index (sensitivity + specificity − 1) was used to quantify the overall trade-off between true positive and false positive rates. The cutoff corresponding to the maximum Youden Index was defined as the optimal operating point, representing the best balance between sensitivity and specificity.

### 2.3 Statistical analysis

Normality of continuous variables was assessed using the Shapiro–Wilk test. For variables meeting the assumption of normality, descriptive statistics were reported as mean ± SD and group comparisons were performed with independent sample t-tests. For variables not meeting normality, results were presented as median [IQR] and compared using Mann–Whitney U tests. Homogeneity of variance for t-tests was verified using Levene’s test. Categorical variables were summarized as frequencies and percentages, with inter-group comparisons conducted using chi-square tests or Fisher’s exact test when expected cell counts were <5. For multiple comparisons, Bonferroni correction was applied to adjust p-values. All statistical analyses were conducted in Python 3.10 using the following libraries: NumPy (v1.24), Pandas (v1.5), SciPy (v1.9), and Statsmodels (v0.13). Data visualization was performed with Matplotlib (v3.6) and Seaborn (v0.12) (18).

## 3 Results

### 3.1 High-grade cytokine release syndrome emerges as the important predictor of clinical deterioration in COVID-19 patients

In the retrospective study of COVID-19, a total of 496 patients were enrolled, consisting of 286 patients from the Wuhan cohort and 210 patients from the Jinan cohort. The patients were categorized based on the disease progression after admission (Improved group vs. Aggravated group. **Supplementary Table S2**). The Aggravated group exhibited a higher prevalence of abnormal coagulation parameters (78.9% vs. 61.0%, p < 0.001) and high-grade CRS compared to the Improved group (91.2% vs. 19.9%, p < 0.001).

**Figure 1:**
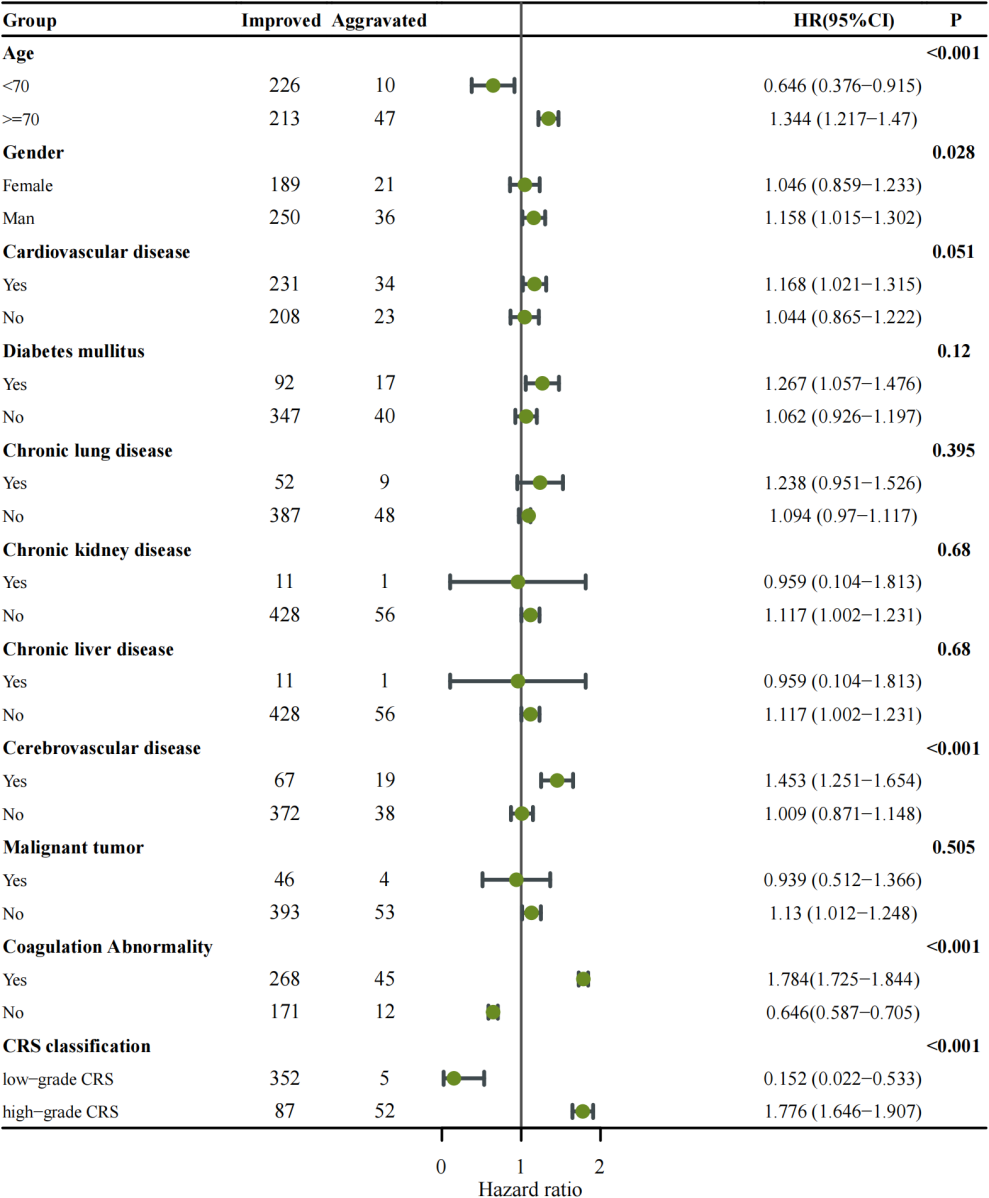
Forest plot showing subgroup analysis of disease progression among COVID-19 patients categorized as Improved versus Aggravated. Each subgroup (age, gender, comorbidities, coagulation abnormality, and CRS classification) was analyzed using Cox proportional hazards regression to estimate the hazard ratio (HR) with corresponding 95% confidence intervals (CIs). The number of patients in each subgroup is indicated in the Improved and Aggravated columns. Points represent HR estimates, and horizontal lines denote 95% CIs. The vertical solid line indicates the null value (HR = 1). Subgroups with p < 0.05 are highlighted in bold. An HR > 1 indicates an increased risk of disease aggravation, whereas HR < 1 suggests a protective effect.

To further investigate the impact of various parameters on disease progression, subgroup analyses with a Forest Plot were performed based on the median values of each parameter or clinical classification standards (**Figure 1**). The Aggravated group was predominantly represented by the following subgroups: age ≥ 70 years (HR: 1.344, 95% CI: 1.217–1.470, p < 0.001), medical history of cerebrovascular disease (HR: 1.453, 95% CI: 1.251–1.654, p < 0.001), abnormal coagulation parameters (HR: 1.784, 95% CI: 1.725–1.844, p < 0.001) and patients with high-grade CRS (HR: 1.776, 95% CI: 1.646–1.907, p < 0.001). Among these, high-grade CRS and abnormal coagulation parameters are major parameters contributing to the aggravation of COVID-19.

### 3.2 Analysis of key risk parameters for high-grade CRS incidence

Given the critical role of high-grade CRS in clinical deterioration, we performed further analyses to identify predictors of severe CRS progression. All the patients were divided into two groups based on their CRS severity (High-grade vs. Low-grade), and a correlation analysis of clinical parameters was performed (**Table 1**). The results indicated that patients with High-grade CRS were significantly older (mean age: 74.76 vs. 67.18 years, p = 0.001) and predominantly male (65.5% vs. 54.6%, p = 0.036). Additionally, patients with High-grade CRS exhibited faster respiratory rates (21.13 vs. 20.55 times/min, p = 0.003), lower SpO_2_ levels (81.15% vs. 95.94%, p = 0.001), and lower blood pressure (112.11/66.61 vs. 131.72/79.85 mmHg, p = 0.001). Compared to Low-grade CRS patients, High-grade CRS patients showed significantly altered coagulation parameters, characterized by higher levels of D-Dimer (3.03 vs. 1.03 μg/ml, p < 0.001), INR (1.18 vs. 1.09, p < 0.001), FDP (11.10 vs. 3.91 μg/ml, p < 0.001), as well as prolonged TT (16.10 vs. 15.80 s, p = 0.008) and PT (13.70 vs. 12.40 s, p < 0.001). These findings suggest that coagulation abnormalities, respiratory and circulatory dysfunction are correlated with the progression of CRS.

**Table 1:** Patient characteristics, comorbidities, monitoring parameters and outcomes of COVID-19 cohorts stratified by CRS Grade (n = 496).

Variable	Total	Low-grade CRS	High-grade CRS	P-value ^a^
Total	496	357 (72.0%)	139 (28.0%)	
Age (year)	69.31 (25.00-99.00)	67.18 (25.00-99.00)	74.76 (40.00-95.00)	**0.001**
Gender (male%)	286 (57.7%)	195 (54.6%)	91 (65.5%)	**0.036**
Respiratory rate (times per minute)	20.70(0.00-40.00)	20.55 (14.00-33.00)	21.13 (0.00-40.00)	**0.003**
Oxygen saturation (%)	91.97 (0.00-100.00)	95.94 (80.00-100.00)	81.15 (0.00-100.00)	**0.001**
Heart rate (times a minute)	86.69 (0.00-155.00)	87.00 (55.00-134.00)	85.83(0.00-155.00)	0.403
Systolic pressure (mmHg)	126.48 (0.00-176.00)	131.72 (90.00-169.00)	112.11 (0.00-176.00)	**0.001**
Diastolic pressure (mmHg)	76.33 (0.00-156.00)	79.85 (56.00-156.00)	66.61 (0.00-139.00)	**0.001**
Outcomes (%)					
Improved	439 (88.5%)	352 (98.6%)	87 (62.6%)	**<0.001**
Aggravated	57 (11.5%)	5 (1.4%)	52 (37.4%)	
Coagulation parameters					
PT(s)	12.70 (11.93-13.68)	12.40 (11.80-13.20)	13.70 (12.50-15.30)	**<0.001**
INR	1.11 (1.04-1.20)	1.09 (1.03-1.16)	1.18 (1.09-1.32)	**<0.001**
FIB(g/L)	3.46 (2.66-4.60)	3.41 (2.66-4.46)	3.63 (2.73-4.68)	0.640
APTT(s)	29.80 (27.30-33.20)	29.65 (27.60-32.40)	30.20 (27.10-34.20)	0.304
TT(s)	15.90 (14.90-16.80)	15.80 (14.90-16.60)	16.10 (15.10-17.70)	**0.008**
DD(μg/ml)	1.16 (0.66-2.75)	1.03 (0.61-1.51)	3.03 (1.03-6.13)	**<0.001**
FDP(μg/ml)	4.80 (3.00-9.70)	3.91 (2.70-5.80)	11.10 (5.34-24.20)	**<0.001**

Categorical data are shown as count (%). Numeric data are presented as mean (range) or median (interquartile ranges).

a Categorical data: P-values arise from chi-square test. Values in bold formatting are statistically significant (p < 0.05).

Numeric data: P-values arise from Mann-Whitney-Wilcoxon test. Values in bold formatting are statistically significant (p < 0.05).

CRS, Cytokine Release Syndrome; PT, prothrombin time; INR, international normalized ratio; FIB, fibrinogen; APTT, activated partial thromboplastin time; TT, thrombin time; D-D, D-dimer; FDP, fibrinogen degradation products.

**Figure 2:**
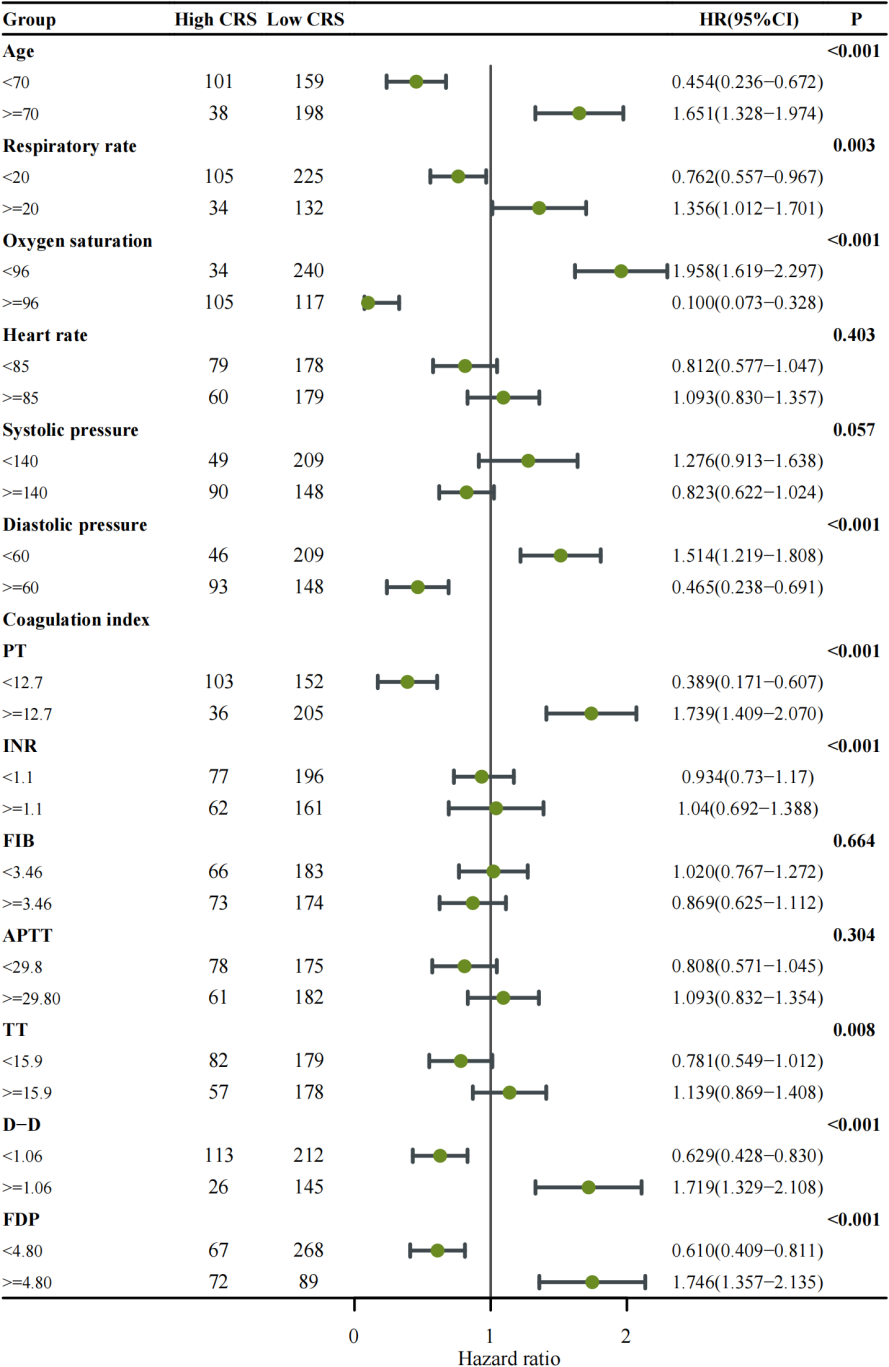
Forest plot showing subgroup analysis of cytokine release syndrome (CRS) classification among patients with COVID-19. Each clinical subgroup—including respiratory rate, oxygen saturation, and coagulation-related indices (PT, INR, FIB, APTT, TT, D-Dimer, and FDP)—was analyzed using Cox proportional hazards regression to estimate the hazard ratio (HR) and corresponding 95% confidence interval (CI). The number of patients within each subgroup is indicated in the table columns. Points represent HR estimates, and horizontal lines denote 95% CIs; the vertical solid line marks the null reference (HR = 1). Subgroups with p < 0.05 are highlighted in bold. An HR > 1 indicates a higher risk of developing high-grade CRS, whereas HR < 1 suggests a lower risk or protective effect.

Further Subgroup analysis with Forest Plot explored the impact of the aforementioned factors on the incidence of High-grade CRS (**Figure 2**). The results revealed that the High-grade CRS was significantly associated with the following factors: older age (≥70 years) (HR = 1.651, 95% CI: 1.328–1.974, p < 0.001), respiratory rate ≥ 20 breaths per minute (HR = 1.356, 95% CI: 1.021–1.701, p = 0.003), oxygen saturation < 96% (HR = 1.958, 95% CI: 1.619–2.297, p < 0.001), and diastolic pressure < 60 mmHg (HR = 1.514, 95% CI: 1.219–1.808, p < 0.001). Notably, coagulation parameters also showed significant associations with the incidence of High-grade CRS, including PT ≥ 12.7 seconds (HR = 1.739, 95% CI: 1.409–2.070, p < 0.001), D-dimer ≥ 1.06 μg/ml (HR = 1.719, 95% CI: 1.329–2.108, p < 0.001), and FDP ≥ 4.8 mg/L (HR = 1.746, 95% CI: 1.357–2.135, p < 0.001). These findings provide crucial insights for the development of a predictive model for the incidence of High-grade CRS.

### 3.3 Development and optimization of the XGBoost model for predicting high-grade CRS

**Figure 3:**
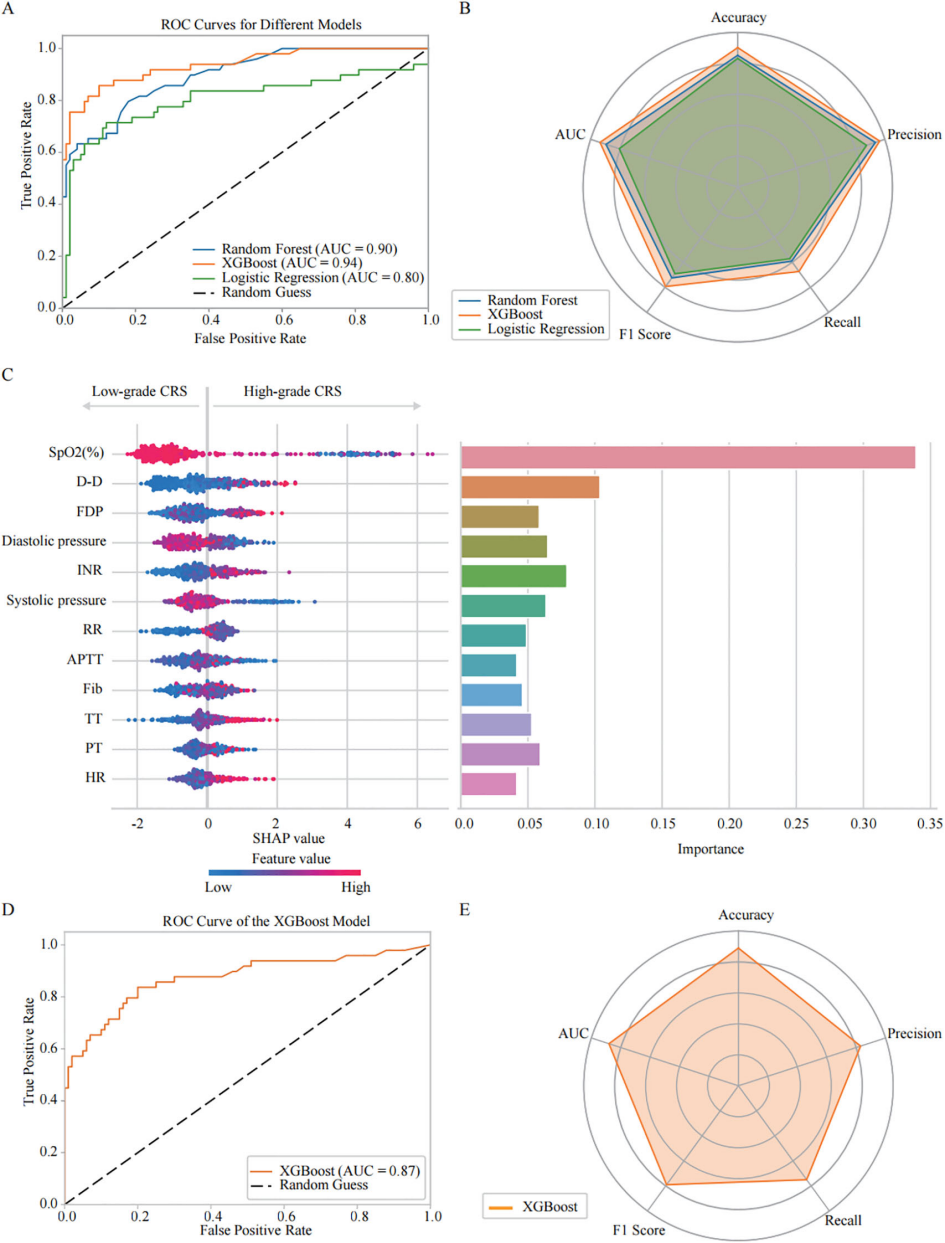
Comparative analysis of machine learning models: predictive performance and feature importance for COVID-19 CRS severity progression. **(A)** Receiver Operating Characteristic (ROC) curves illustrate the predictive performance of three models: Random Forest (AUC = 0.90), XGBoost (AUC = 0.94), and Logistic Regression (AUC = 0.80). **(B)** Radar plot summarizing model performance across five evaluation metrics: precision, accuracy, recall, F1 score, and AUC. Random Forest: precision (0.94), accuracy (0.85), recall (0.59), F1 score (0.73), AUC (0.90); XGBoost: precision (0.96), accuracy (0.90), recall (0.67), F1 score (0.79), AUC (0.94); Logistic Regression: precision (0.88), accuracy (0.83), recall (0.57), F1 score (0.69), AUC (0.80). Among the three models, XGBoost demonstrated the highest overall performance. **(C)** SHAP analysis of the XGBoost model evaluating feature importance for CRS classification. The left figure shows the SHAP summary plot, where the y-axis lists input features ranked by their mean absolute SHAP values across all samples—features with higher rankings contribute more strongly to the overall model output. The x-axis represents SHAP values, reflecting both the direction and magnitude of each feature’s influence on predicting high-grade CRS. Each dot corresponds to an individual patient sample, with color indicating the actual feature value (red = high, blue = low). The right bar chart presents the XGBoost feature importance, calculated based on the average information gain across all decision trees. **(D)** ROC curve analysis showing the XGBoost model’s predictive power for CRS severity classification in the validation cohort (AUC = 0.87). **(E)** Radar plot presenting XGBoost model performance across evaluation metrics: accuracy (0.89), precision (0.83), recall (0.75), and F1 score (0.79).

Then, we developed and comparatively evaluated three machine learning models - Random Forest, XGBoost, and Logistic Regression - using clinical datasets to analysis and feature importance of the parameters associated with High-grade CRS. The ROC curve visually illustrated the discriminative ability of each model in disease aggravation (**Figure 3A**). The results indicated that the XGBoost model outperformed the others, achieving an AUC of 0.94. In contrast, the AUC values for the Random Forest and Logistic Regression models were 0.90 and 0.80, respectively. Radar Charts were also employed for multi-dimension analysis of the models’ performance, including accuracy, precision, recall, F1 score, and AUC. The results showed that XGBoost consistently demonstrated superior performance across all the indicators, particularly in recall (0.67), F1 score (0.79) and precision (0.96) (**Figure 3B**). Based on these findings, we selected XGBoost as the primary model for consequent analysis.

We then optimized the XGBoost algorithm with GridSearchCV. The hyperparameters were tuned as follows: a learning rate of 0.01, 100 estimators, and a maximum depth of 4, along with other specified values. This optimization allowed us to conduct a more in-depth analysis of the key features associated with CRS aggravation. The SHAP Value Plots and Feature Importance Bars revealed the relative contributions of each parameter to the optimized XGBoost model’s predictions (**Figure 3C**). The Feature Importance Bar chart (on the right) scored the various features based on their importance in the model, while the SHAP value plot (on the left) further illustrated the direction and intensity of each feature’s impact on predicting CRS aggravation. All SHAP values were computed on a unified scale, allowing direct comparison of the relative contribution of different features to the model output. The importance scores were normalized so that their total equals 1, representing the proportional contribution of each feature to the overall model performance. The results indicate that SpO_2_ (%) is the most critical predictor for the occurrence of High-grade CRS, with lower SpO_2_ (%) showing the strongest association with High-grade CRS incidence. Among the coagulation parameters, D-dimer, INR and FDP were also identified as important factors in predicting High-grade CRS. Elevated levels of D-dimer and FDP, higher INR strongly correlate with the incidence of High-grade CRS. Additionally, blood pressure, particularly diastolic pressure, was also found to promote the development of High-grade CRS. Other factors were also found to be associated with High-grade CRS, including RR, APTT, Fib, TT, PT, and HtR. These features provide valuable predictive insights for the incidence of High-grade CRS.

Based on the feature importance and contribution ranking of the relevant clinical parameters identified by the optimized XGBoost model, we selected the top four parameters, including SpO_2_, D-dimer, diastolic pressure, and INR, to simplify the prediction model for High-grade CRS, making it more suitable for clinical practice. The performance evaluation of the simplified XGBoost model indicated that AUC reached 0.87 (**Figure 3D**). The Radar Chart demonstrated that the model maintained high accuracy (0.89), precision (0.83), recall (0.75), and F1 score (0.79) (**Figure 3E**), which suggest the simplified XGBoost model retains a high level of predictive efficiency in high-grade CRS prediction.

### 3.4 Validation of simplified XGBoost model with CAR-T therapy cohort

**Figure 4:**
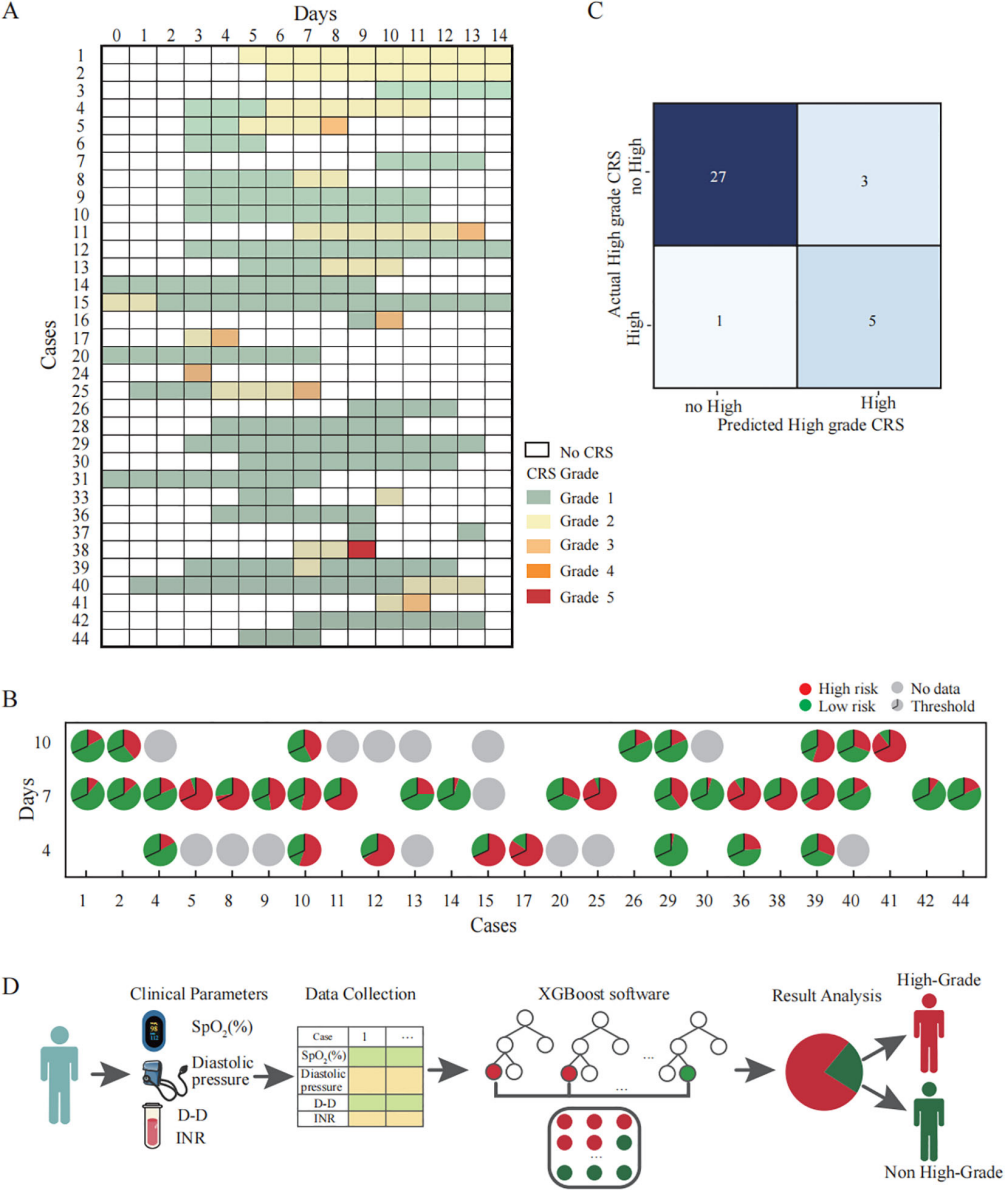
Validation and clinical application of the simplified XGBoost model for predicting high-grade CRS in CAR-T therapy cohorts. **(A)** Clinical records of patients who developed CRS after CAR-T infusion. Each row corresponds to an individual patient, and the columns represent the day after CAR-T infusion. The color of the squares represents the grade of CRS. **(B)** The probability of developing high-grade CRS at each time points predicted by a simplified XGBoost model. Each pie chart represents the model’s predicted probability for an individual patient at a specific time point (Days 4, 7, and 10 after CAR-T infusion). The entire circle corresponds to 100% of the prediction probability: The red segment and the green segment respectively represent the occurrence probabilities of high grade CRS and non-high grade CRS. The black line within each pie denotes the classification threshold (cutoff = 0.68) derived from the training dataset. When the red portion exceeds this threshold, the model predicts that the patient is likely to develop high-grade CRS. **(C)** Confusion matrix illustrating the predictive performance of the simplified XGBoost model for identifying high-grade CRS. Each cell represents the number of cases where the predicted classification agrees or disagrees with the actual clinical outcome. The model correctly identified 5 of 6 patients with high-grade CRS (true positives) and 27 of 30 patients with no-high-grade CRS (true negatives). **(D)** Schematic illustration of the clinical workflow for applying the simplified XGBoost model to predict the risk of high-grade CRS. First, Clinical parameters are collected as model inputs. Subsequently, the XGBoost model to calculate the probability score. Finally, the model output is visualized to classify patients into predicted high-grade CRS (red) and non–high-grade CRS (green) groups.

Furthermore, we included clinical observation data from 45 CAR-T therapy patients as the test dataset. Among these patients, 9 cases were hematological tumors and 36 cases were solid tumors. During the clinical observation period, 8 patients experienced high-grade CRS (17.78%), 26 patients experienced only low-grade CRS (57.78%), and 11 patients did not develop CRS at any level (24.45%) (**Figure 4A**). We analyzed the correlation between clinical parameters and high-grade CRS, which showed no significant associations between the development of high-grade CRS and clinical parameters such as age, gender, ECOG score, prior treatment lines, medical history, or CAR-T treatment targets (**Table 2**). We collected clinical test results during episodes of low-grade CRS, including four key parameters: SpO_2_, D-dimer, diastolic pressure, and INR. A total of 36 complete data sets were collected from 25 CRS patients across three longitudinal time points, including 6 sets obtained from patients experiencing high-grade CRS (**Figure 4B**). We applied the simplified XGBoost model to assess the risk of high-grade CRS with the dataset. Based on the risk analysis for high-grade CRS, we categorized patients with a risk of ≥68% as high risk and those with <68% as non-high risk. The efficiency of the model in predicting the risk of high-grade CRS was analyzed in conjunction with actual clinical observations and was displayed using a confusion matrix (**Figure 4C**). Among the 6 cases of high-grade CRS, the model correctly predicted 5 cases and incorrectly predicted 1. For patients who did not develop high-grade CRS, the model correctly predicted 27 cases and incorrectly predicted 3. The sensitivity and specificity of the simplified XGBoost model in predicting the occurrence of high-level CRS are 0.83 and 0.90, respectively. We packaged the simplified XGBoost model into a software tool (crs_predictor) for clinical practice (Supplemental. The CRS_predictor software). For cancer patients undergoing CAR-T therapy with a risk of high-grade CRS, the four monitored parameters (SpO_2_, D-dimer, diastolic pressure, and INR) can be used in conjunction with the software to efficiently predict the risk of high-grade CRS (**Figure 4D**).

**Table 2:** The Characteristics, comorbidities, and monitoring parameters of the CAR-T therapy cohort (n = 45).

Variable	Total	No or low-grade CRS	High-grade CRS	P-value ^a^
Total	45	37 (82.2%)	8 (17.8%)	
Age, years				0.945
< 58	23 (51.1%)	19 (51.3%)	4 (50.0%)	
≥58	22 (48.9%)	18 (48.6%)	4 (50.0%)	
Gender				0.136
Male, No. (%)	23 (51.1%)	17 (45.9%)	6 (75.0%)	
Female, No. (%)	22 (48.9%)	20 (54.1%)	2 (25.0%)	
ECOG, No. (%)				0.270
0-1	30 (66.7%)	26 (70.3%)	4 (50.0%)	
2	15 (33.3%)	11 (29.7%)	4 (50.0%)	
No. of previous lines, n (%)				0.100
2	12 (15.6%)	8 (8.1%)	4 (50.0%)	
≥3	33 (73.3%)	29 (78.4%)	4 (50.0%)	
Tumor type, No. (%)				0.697
Solid tumor	36 (80%)	30 (81.1%)	6 (75.0%)	
Hematologic tumors	9 (20%)	7 (18.9%)	2 (25.0%)	
Medical history (%)				
Cardiovascular disease	10 (22.2%)	5 (13.5%)	5 (62.5%)	0.003
Diabetes mellitus	6 (13.3%)	4 (10.8)	2 (25%)	0.284
Chronic lung disease	1 (2.2%)	1 (2.7%)	0 (0.0%)	0.638
Chronic liver disease	1 (2.2%)	1 (2.7%)	0 (0.0%)	0.638
Chronic kidney disease	1 (2.2%)	1 (2.7%)	0 (0.0%)	0.638
Cerebrovascular disease	6 (13.3%)	3 (8.1%)	3 (37.5%)	0.027
Targets				0.465
CD19	9 (20.0%)	7 (18.9%)	2 (25.0%)	
CEA	29 (64.4%)	25 (67.6%)	4 (50.0%)	
MSLN	2 (4.4%)	2 (5.4%)	0 (0.0%)	
ROR1	5 (11.1%)	3 (8.1%)	2 (25%)	

a Categorical data: P-values arise from chi-square test. Values in bold formatting are statistically significant (p < 0.05).

CRS, Cytokine Release Syndrome; ECOG, Eastern Cooperative Oncology Group; CEA, carcino-embryonic antigen; MSLN, mesothelin; ROR1, the receptor tyrosine kinase-like orphan receptor 1.

## 4 Discussion

This study employed the XGBoost algorithm to construct a predictive model for assessing the risk of progression to high-grade CRS following its onset, thereby offering critical evidence for evaluating the safety and efficacy of CAR-T therapy in cancer patients. CRS is characterized by a cascade of cytokine activation and immune cell hyperactivation, which can be triggered by infection, various immunotherapeutic, malignant tumors, autoimmunity (19). COVID-19 patients who develop CRS exhibit similar clinical features and share a common pathophysiological mechanism with CAR-T recipients, thereby serving as a valuable surrogate dataset for model development, given the scarcity of large-scale clinical data from CAR-T-treated cancer patients. Previous research has demonstrated that fatalities in severe COVID-19 are associated with elevated levels of circulating pro-inflammatory cytokines (20) and that high-grade CRS significantly correlates with COVID-19 severity. Furthermore, several studies confirm that severe immune dysregulation during COVID-19 progression, along with inflammasome-associated cytokines such as IL-1β and IL-18, is significantly associated with vascular damage (5, 21). Coagulopathy—characterized by elevated D-dimer levels, prolonged PT, and decreased coagulation proteins—has been shown to predict poor outcomes in severe COVID-19 cases (22–24). Our study highlights that abnormal coagulation parameters may serve as early diagnostic biomarkers. Notably, these characteristics closely resemble those observed following CAR-T cell infusion. These findings regarding the pivotal role of CRS in COVID-19 progression further support the utility of this dataset as a valuable resource for modeling CRS progression.

Subsequently, clinical big data from COVID-19 patients with CRS were analyzed, enabling the identification of key clinical parameters associated with the incidence of high-grade CRS, including SpO_2_, D-dimer, diastolic pressure, and INR. A machine learning-based decision tree model was developed using these parameters, which showed high predictive performance in estimating the risk of high-grade CRS in CAR-T–treated patients.

The XGBoost model has emerged as a powerful tool for predicting disease progression, with several advantages that informed its selection in this study. First, XGBoost is highly effective in handling high-dimensional datasets, which makes it particularly suitable for medical data analysis. For example, XGBoost has demonstrated superior performance in predicting the progression and metastasis of thyroid cancer compared to traditional models (25). Additionally, its efficient data processing capabilities enable rapid training and prediction, showcasing its versatility and reliability across various medical fields (26). In recent years, XGBoost has also shown promising potential in predicting severe outcomes of acute diseases, such as predicting respiratory failure in COVID-19 patients (27). Another advantage is the interpretability of XGBoost’s results, which enhances clinical understanding and facilitates the application of its predictions in practice (28). In the present study, the XGBoost model played a pivotal role in identifying key parameters associated with high-grade CRS incidence. By ranking feature importance and contributions, it yielded valuable insights into the most influential predictors. Specifically, key predictive indicators—such as SpO_2_, D-Dimer, diastolic blood pressure, and INR—were identified as potentially significant contributors to high-grade CRS development and warrant further investigation.

The pathophysiological mechanisms underlying CRS involve several intertwining processes, including inflammatory cytokine production, endothelial activation, and coagulopathy. An exaggerated interplay between these processes create a vicious cycle that damages organs and vasculature (29). One central mechanism of CRS is endothelial cell activation, manifested through increased endothelial permeability and a pro-inflammatory state (30), concurrently accompanied by loss of vascular integrity and a transition to a prothrombotic phenotype (31).

D-dimer, a fibrin degradation product, serves as a biomarker for coagulation and fibrinolysis and is commonly used in laboratory tests. Elevated D-dimer is a well-established predictor of severe COVID-19 manifestation—including thromboembolism (32), acute respiratory distress syndrome (33), and mortality (22, 34)—and has also been implicated in predicting CRS development (35). In anti-CD19 CAR-T cell therapy, D-dimer elevation correlates with endothelial injury and CRS severity (30), mirroring similar pathophysiological mechanisms observed in COVID-19 (36). Mechanistic studies have shown that D-dimer can induce monocytes to release pro-inflammatory cytokines such as IL-6, thereby exacerbating the inflammatory response in CRS (37). This hypercoagulable state arises from enhanced thrombin generation coupled with suppressed fibrinolysis—processes exacerbated during CRS that resemble those observed in sepsis and disseminated intravascular coagulation (DIC) (38). Collectively, these findings suggest that elevated D-dimer levels represent a distinctive immunological hallmark of disease progression (39), offering valuable insights for assessing CRS severity.

The INR is a critical clinical marker for assessing coagulation status. An elevated INR reflects underlying coagulation dysfunction. Elevation in INR correlates with the severity of dysfunction and endothelial injury, illustrating how coagulopathy is integrally involved in CRS pathogenesis. As CRS develops, inflammatory activation further disrupts coagulation pathways, worsening the INR elevation (40). This phenomenon is exemplified in COVID-19, where systemic inflammation induces disseminated intravascular coagulation, often accompanied by characteristic INR elevation (41). Previous studies have demonstrated that an elevated INR retains predictive value for CRS severity across diverse clinical scenarios, including SARS-CoV-2 infection and therapeutic interventions such as monoclonal antibody treatments (42) and anti-thymocyte globulin administration (43). Therefore, elevated INR may represent a valuable early biomarker for the detection of CRS and associated coagulation disorders.

SpO_2_ and hemodynamic instability play crucial roles in the development and progression of CRS (44). Studies have shown that hypoxemia (45) and diastolic pressure (46, 47) has been identified as an early biomarker for assessing CRS risk in COVID-19 patients. Mechanistically, excessive IL-6 signaling promotes the accumulation of innate and adaptive immune cells in the lungs (48) and amplifies the inflammatory cascade, leading to damage of capillary endothelial and lung epithelial cells (49). Concurrently, systemic inflammation disrupts the coagulation-fibrinolysis balance and impairs hypoxic compensatory mechanisms, exacerbating microthrombosis and worsening hypoxia and tissue injury (20). In summary, CRS-induced endothelial activation increases vascular permeability, and coagulopathy leads to microthrombosis. These alterations collectively compromise pulmonary microcirculation, impair gas exchange, and result in hypoxemia. Furthermore, elevated inflammatory mediators disrupt vascular tone and endothelial function, causing dysregulation of blood pressure and increased vascular resistance, particularly affecting diastolic pressure (50). Therefore, monitoring oxygen saturation and diastolic blood pressure is essential for assessing the severity and prognosis of CRS. Notably, while hypoxemia and hypotension are included as clinical criteria in current CRS grading systems, our predictive model integrates these parameters as continuous physiological variables rather than binary thresholds used for clinical interventions. Through quantitative analysis of their temporal dynamics in patients with low-grade CRS, we identify subclinical deterioration patterns that remain undetected by conventional ordinal grading systems.

There has been notable progress in improving the prediction models for CRS using high-dimensional analysis. Specific inflammatory cytokine profiles, including elevated levels of IL-6 and TNF-α, have been shown to predict the severity of COVID-19 (5, 51). Predictive models for real-time CRS risk assessment have also found applications in oncology settings (52). The XGBoost model, employed in this project using machine learning techniques, highlights the potential of advanced analytical methods in clinical practice, demonstrating notable advantages in risk factor identification and the design of personalized intervention strategies. When the model identifies a high risk of severe CRS (predicted probability≥0.68), it alerts the clinical team to consider initiating a standardized protocol for proactive management. This protocol encompasses intensified clinical monitoring, early readiness for intervention with anti-IL-6 agents or corticosteroids, multidisciplinary team (MDT) evaluation, and advance preparation for potential ICU transfer. Through this integrated management pathway, early recognition and mitigation of toxicity become feasible, thereby enhancing the overall safety and therapeutic efficacy of CAR-T treatment.

One limitation of this study is the small sample size of the validation dataset, which is a common limitation in the current CAR-T therapy research. As most CAR-T cell therapies remain in the clinical trial stage (53), heterogeneous clinical observation data from different research centers, the sample size of the validation data set is limited to a certain extent. Another important limitation concerns the influence of treatment interventions. We observed three cases in which the model predicted high-grade CRS, whereas the actual clinical grade was low. Notably, all of these patients had received glucocorticoid therapy. This finding implies that the model’s “false-positive” predictions may, in fact, reflect patients at genuine high risk whose severe reactions were effectively prevented by timely treatment. Such an interaction between predictive output and therapeutic intervention introduces bias into retrospective validation and constitutes a critical limitation of the study. Consequently, prospective validation in treatment-naïve settings will be essential to accurately determine the model’s independent predictive performance.

In conclusion, this study successfully addresses the critical challenge of predicting high-grade CRS in CAR-T therapy by developing a robust machine learning-based risk-assessment algorithm. The validated XGBoost model, leveraging key predictors (SpO_2_, D-dimer, diastolic BP, INR) identified through comprehensive analysis, demonstrates strong predictive capability and represents a significant advancement over existing approaches. Notably, our predictive model—originally developed using data from COVID-19 patients and subsequently validated in CAR-T therapy—may also hold potential for broader application across other CRS-associated contexts, such as bispecific antibody (BsAb) therapies and immune checkpoint inhibitors (ICIs). These therapeutic modalities share core immunopathological mechanisms, including T-cell hyperactivation, cytokine release, endothelial dysfunction, and coagulopathy, as documented in prior studies (54, 55). Such overlapping biological processes suggest that our biomarker-driven framework could be adapted to anticipate and manage CRS across diverse forms of immunotherapy.

The primary future perspective centers on translating this validated predictive model into routine clinical practice. Integrating this tool into CAR-T treatment protocols is paramount, as it enables early identification of high-risk patients, facilitating timely interventions to mitigate severe CRS and ultimately improve patient safety and outcomes. Further validation across larger, multi-center CAR-T cohorts will solidify its generalizability. The deployment of this predictive algorithm holds promise for personalizing CAR-T therapy and enhancing its therapeutic index.

## Data Availability

The raw data supporting the conclusions of this article will be made available by the authors, without undue reservation.
